# The effects of repetitive transcranial magnetic stimulation on the whole-brain functional network of postherpetic neuralgia patients

**DOI:** 10.1097/MD.0000000000016105

**Published:** 2019-06-21

**Authors:** Qian Pei, Zhizheng Zhuo, Bin Jing, Qianqian Meng, Xiangyu Ma, Xiao Mo, Han Liu, Wei Liang, Jiaxiang Ni, Haiyun Li

**Affiliations:** a Beijing Jishuitan Hospital; b Department of Pain Management, Xuanwu Hospital Capital Medical University; c School of Biomedical Engineering, Capital Medical University, Beijing, China.

**Keywords:** functional magnetic resonance imaging, functional network, postherpetic neuralgia, repetitive transcranial magnetic stimulation

## Abstract

The effects of repetitive transcranial magnetic stimulation (rTMS), the clinical treatment for postherpetic neuralgia (PHN), on whole-brain functional network of PHN patients is not fully understood.

To explore the effects of rTMS on the whole-brain functional network of PHN patients.

10 PHN patients (male/female: 5/5 Age: 63–79 years old) who received rTMS treatment were recruited in this study. High-resolution T1-weighted and functional Magnetic Resonance Imaging (fMRI) were acquired before and after 10 consecutive rTMS sessions. The whole-brain functional connectivity networks were constructed by Pearson correlation. Global and node-level network parameters, which can reflect the topological organization of the brain network, were calculated to investigate the characteristics of whole-brain functional networks. Non-parametric paired signed rank tests were performed for the above network parameters with sex and age as covariates. *P* < .05 (with FDR correction for multi-comparison analysis) indicated a statistically significant difference. Correlation analysis was performed between the network parameters and clinical variables.

The rTMS showed significant increase in characteristic path length and decrease of clustering coefficient, global, and local efficiency derived from the networks at some specific network sparsity, but it showed no significant difference for small-worldness. rTMS treatment showed significant differences in the brain regions related to sensory-motor, emotion, cognition, affection, and memory, as observed by changes in node degree, node betweenness, and node efficiency. Besides, node-level network parameters in some brain areas showed significant correlations with clinical variables including visual analog scales (VAS) and pain duration.

rTMS has significant effects on the whole-brain functional network of PHN patients with a potential for suppression of sensory-motor function and improvement of emotion, cognition, affection, and memory functions.

## 1 Introduction

Clinically, postherpetic neuralgia (PHN) is defined as a type of chronic neuropathic pain due to herpes zoster infection. It is characterized as a sharp, burning or stabbing pain which is usually associated with irreparable damage to the peripheral nervous system.^[1–3]^ It can affect the quality of life of patients because of loss of productivity and the associated cost of treatment.^[4]^ Currently, the general clinical treatment is pharmacological intervention which might lead to considerable risk of side effects especially from long-term drug use.^[5]^ Thus, a new clinical treatment, repetitive transcranial magnetic stimulation (rTMS), which can noninvasively send magnetic stimulation to a specific region of the brain, has been shown to effectively relieve pains in PHN patients with fewer side effects.^[6]^

Previous studies have investigated the underlying central neural mechanism of chronic neuropathic pain including PHN. A diffusion kurtosis imaging (DKI) study found anatomical gray matter damage from PHN within some brain regions including bilateral insula, superior temporal gyrus, middle frontal gyrus and occipital lobe, cerebellum anterior lobe, thalamus (THA), caudate (CAU), and parahippocampal gyrus.^[7]^ Besides the structural alterations, the functional changes seen in the brain of PHN patients have drawn a lot of clinical attentions. Compared to normal controls, several brain regions including the brainstem, THA, limbic system, temporal lobe, prefrontal lobe, and cerebellum showed abnormal ReHo (regional homogeneity) and fractional amplitude of low-frequency fluctuations (fALFF) values in PHN patients. These findings indicated that the functional changes of PHN patients were not just limited to the pain modulation circuitry but extend to the areas in charge of affective processes and emotional activity.^[8]^ Nevertheless, these studies were only focused on the changes in isolated local brain region. As an integrated neural information exchange system, the brain functional connectivity network that reflects the brain functional changes at a whole-brain level can be used to find the underlying information exchange pattern in PHN patients. In fact, altered interhemispheric intrinsic connectivity related to the dorsolateral prefrontal cortex, the precuneus, and posterior cingulate cortex has been reported in PHN patients.^[9]^ Almost all Magnetic Resonance Imaging (MRI) studies to date focused on the underlying neural changes of PHN patients compared to normal volunteers, but rarely on the structural or functional alterations after clinical treatments.

Recently, rTMS has been adopted for the treatment of neurological and psychiatric disorders in clinics.^[6]^ Some studies show that rTMS (≥5 Hz) targeting primary motor cortex (M1) has pain-relieving effects in neuropathic pain patients.^[6,10]^ The underlying possible mechanism is thought to be the modulation of the neural activities in the pain, emotion, cognition, and affection related structures.^[11]^ It could be possible that the stimulus might act within the M1, which could be regarded as an entry point, and then modulate the other remote or deep brain regions through subcortical fibers.^[12]^ Accumulated evidence demonstrated that rTMS not only affects the target areas but also other distant brain regions. Therefore, a whole-brain level exploration of function network to find all the potential affected neural circuitries of rTMS is essential and important.

Although several studies have reported the effect of rTMS on some types of neuropathic pain, only a few have described its effect on the whole-brain intrinsic functional network in PHN patients. According to the PHN clinical symptom and previous evidence, the whole-brain intrinsic functional network in PHN patients might change after the rTMS treatment, which could be measured by resting-state fMRI analysis.

## 2 Materials and methods

### 2.1 Subjects

Twelve patients (5 males and 7 females, aged from 63 to 79 years old, all right-handed) were recruited in this study from Sep 2017 to Jan 2018 in Xuanwu Hospital, Beijing, China. All patients were informed of the purpose of this study and the study was approved by the Ethics Committee of Xuanwu Hospital.

In this study, the inclusion criteria were:

1. persistent pain caused by herpes zoster infection lasting for more than a month; and
2. receiving the conventional drug therapy but with little effect on pain intensity.

Patients were excluded if:

1. other neural or physical disorders, diabetes or history of brain surgery;
2. unable to undergo MRI examination due to mental disorders such as claustrophobia or with metal implant; or
3. with poor quality of MRI images.

Two patient were excluded due to incomplete MRI examination and imaging quality issues. Finally, 10 patients included in this study (Table 1).

**Table 1:**
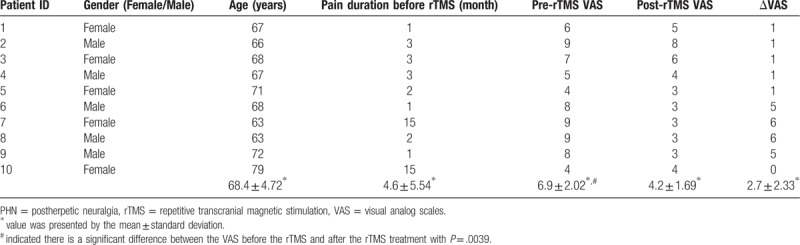
Demographic information of the patients included in this study.

### 2.2 Repetitive transcranial magnetic stimulation

All patients received 10 consecutive rTMS sessions. The stimulation target was located on the contralateral primary motor cortex of the lesion location. The rTMS parameters were: 300 repetitive 1-second pulses with 5-Hz frequency and interval of 2.5 seconds between trains, for a total of 1500 pulses per session; the stimulation intensity was 80% of the resting motor threshold. rTMS was performed using a transcranial magnetic stimulator (CCY-III, Wuhan Yiruide Medical). All patients underwent MRI examinations twice before and after the 10 consecutive rTMS sessions. The pain intensity was evaluated by pre- and post-treatment VAS and pre-treatment pain duration were recorded.

### 2.3 Image acquisition

All images were acquired using a 3-T scanner (Ingenia, Philips, The Netherlands) and a 15-channel head coil. High-resolution 3D T1-weighted protocol and resting-state fMRI protocol were used for image acquisition. For 3D T1-weignted imaging, the protocol parameters were: 3D FFE acquisition, flip angle = 15^∘^, TR/TE = 4.5 ms/2.1 ms, FOV = 240 mm × 240 mm, image resolution = 1 mm × 1 mm, slice thickness = 1 mm with no slice gap, matrix size = 240 × 240, Slice number = 160, Sense factor = 1.3, phase encoding direction = AP, NSA = 1, scan time = 4 minutes 23 seconds. For fMRI imaging, the protocol parameters were as follows: Multi-slice FE-EPI acquisition with SPIR fat suppression, flip angle = 90^∘^, TR/TE = 2000 ms/35 ms, FOV = 230 mm × 230 mm, image resolution = 3 mm × 3 mm, slice thickness = 3.5 mm with no slice gap, matrix size = 76 × 76, slice number = 33, sense factor = 2, phase encoding direction = AP, NSA = 1, scan time = 240 seconds with 120 dynamic volumes. Subjects were asked to keep their eyes closed and think of nothing during the scan. Patients on tegretol or other painkillers were asked to stop taking the drugs 1 week before the MRI examination to avoid drug effects on the fMRI data.

### 2.4 Processing of MRI images

The resting-state fMRI images were preprocessed using Data Processing Assistant for Resting-State fMRI, http://restfmri.net/forum/index.php (DPARSFA). The image preprocessing included:

1. removing the first 10 time points;
2. slice timing and head motion correction;
3. regression of nuisance covariates including realign parameters, mean white matter and CSF signals;
4. linear detrending and band-pass filtering within 0.01 to 0.1 Hz;
5. co-registration of fMRI images to the high-resolution 3D T1 structural images and normalization of 3D T1w structural MRI images into MNI space by non-linear warping;
6. normalization of fMRI images into MNI space by using the same parameters derived from the structural image normalization and simultaneously resampled into 3-mm isotropic voxels;
7. smoothing the normalized fMRI images with a Gaussian kernel of 4 mm full width at half maximum.

Finally, the average time series within each region based on AAL_116 atlas was extracted from the normalized and smoothed fMRI images for the following construction of brain functional connectivity network.

### 2.5 Function connectivity network construction & network parameters calculation

The functional connectivity strength between each pair of brain regions was presented by using bivariates Pearson correlation. Finally, a 116 × 116 weighted connectivity matrix was obtained for each fMRI dataset.

The absolute weighted matrix was used to extract the network parameters, including small-worldness, characteristic path length, clustering coefficient, global and local efficiency, node degree, node betweenness, node efficiency at a series of network sparsity from 0.01 to 0.5 with an interval of 0.01. All calculations were carried out using the GRETNA toolbox.^[13]^

### 2.6 Statistical analysis

For the clinical variables, non-parametric paired signed rank tests were performed, with *P* < .05 indicating a significant value.

For network parameters, small-worldness, characteristic path length, clustering coefficient, global and local efficiency, non-parametric paired signed rank tests were performed at different network sparsity with sex and age as covariates. For node degree, node betweenness and node efficiency, non-parametric paired signed rank test was performed at each node with the averaged values across network sparsity.

Correlation analysis was performed between the network parameters and clinical variables. For all analyses *P* < .05 was considered significant.

## 3 Results

### 3.1 Functional network alteration after rTMS treatment

**Figure 1:**
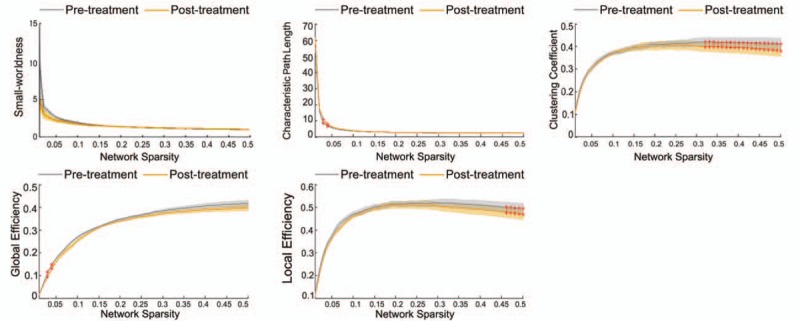
The alteration of small-worldness, characteristic path length, clustering coefficient, global and local efficiency at a series of network sparsity (0.01−0.5 with interval of 0.01). After the rTMS treatment, the small-worldness showed no significant alteration, but the characteristic path length, clustering coefficient, global and local efficiency showed significant alterations at some network sparsity. Red ^∗^ indicate the values showed significant difference corresponding to the specific network sparsity.

After rTMS treatment, we observed a significant increase in the characteristic path length (network sparsity = 0.03 and 0.04) and decrease in the clustering coefficient (network sparsity ≥0.32), global efficiency (network sparsity = 0.03 and 0.04) and local efficiency (network sparsity ≥0.46) derived from the networks at some specific network sparsity but no significant difference for small-worldness (Fig. 1).

**Figure 2:**
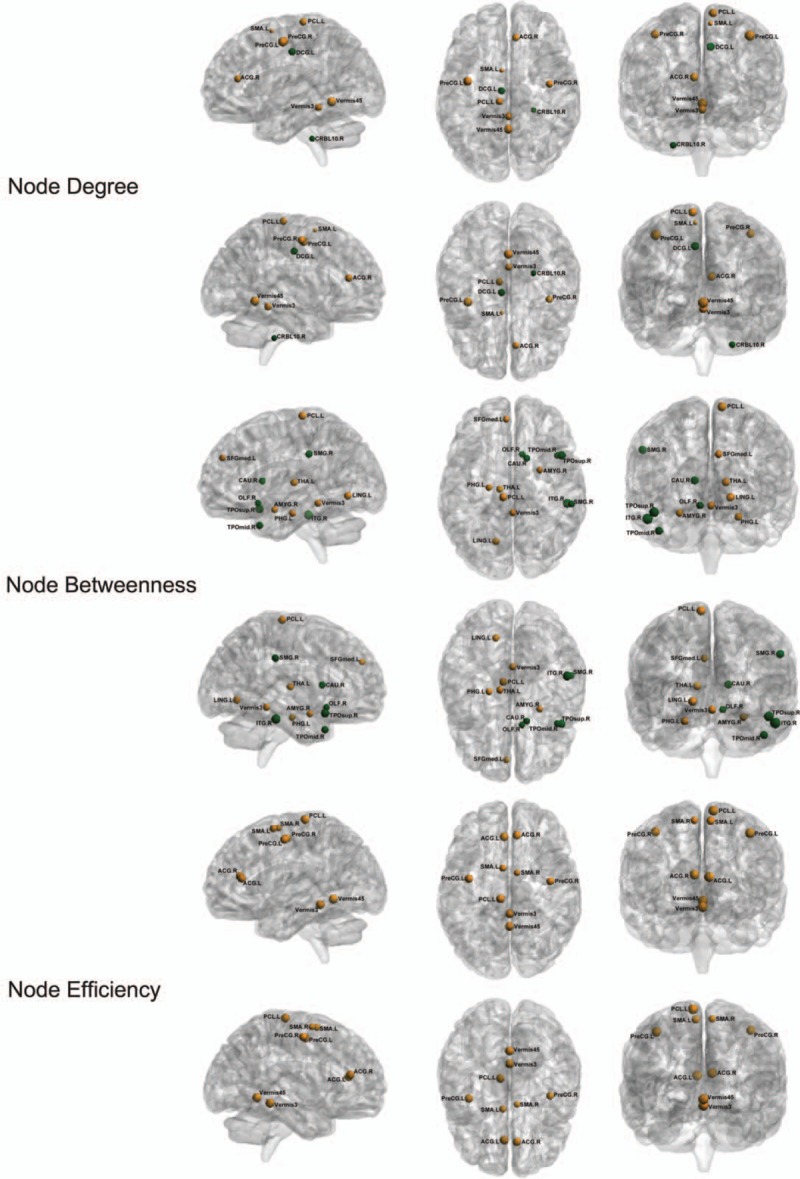
The alteration of node degree, node betweenness and node efficiency. Increased node is presented in green and decreased node is presented in yellow. The size of the node presented the difference of the pre- and post-rTMS treatment node-level parameters.

A significant increase in node degree was observed in the brain regions of the left middle cingulum gray matter (DCG.L) and right cerebellum 10 gray matter (CRBL10.R), and a decrease in node degree in the brain regions of left precentral grey matter (PreCG.L), right precentral gray matter (PreCG.R), right anterior cingulum gray matter (ACG.R), left supplementary motor area (SMA.L), left paracentral lobule (PCL.L), Vermis3 and Vermis45 (Fig. 2).

Node bewteenness showed significant increase in the brain regions of right supra marginal gray matter (SMG.R), right caudate (CAU.R), right inferior temporal gray matter (ITG.R), right olfactory (OLF.R), right middle temporal pole (TPOmid.R), and superior temporal pole (TPOsup.R), and decrease in the brain regions of PCL.L, left thalamus (THA.L), left lingual (LING.L), left median superior frontal gray matter (SFGmed.L), right amygdala (AMYG.R), left para-hippocampal gray matter (PHG.L) and Vermis3 (Fig. 2).

Node efficiency was significantly decreased in the brain regions of PreCG.L, PreCG.R, left anterior cingulum gray matter (ACG.L), ACG.R, SMA.L, right supplementary motor area (SMA.R), PCL.L, Vermis3, Vermis45. There were no regions that showed increase in Node efficiency (Fig. 2).

### 3.2 Correlation between network parameters and clinical variables

Spearman correlation analysis was performed to explore the relationship of the clinical variables with the global parameters and node-level parameters of the brain regions that showed significant alterations after rTMS.

For small-worldness, characteristic path length, clustering coefficient, global and local efficiency, there was no significant correlation of pre- or post-treatment values with pre-treatment VAS, post-treatment VAS, ΔVAS (pre-treatment VAS minus post-treatment VAS), or pain duration.

For node degree, node betweenness, and node efficiency, we observed some significant correlations (*P* < .05) of the pre-treatment, post-treatment, and difference (pre-treatment minus post-treatment) values with pre-treatment VAS, ΔVAS, or pain duration (Tables 2–4, respectively).

**Table 2:**
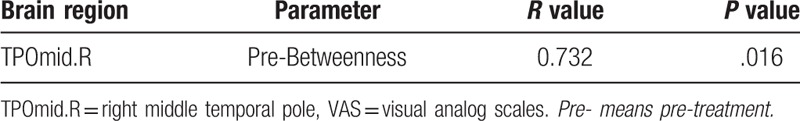
Correlation of node-level parameters with VAS (*P* < .05).

**Table 3:**
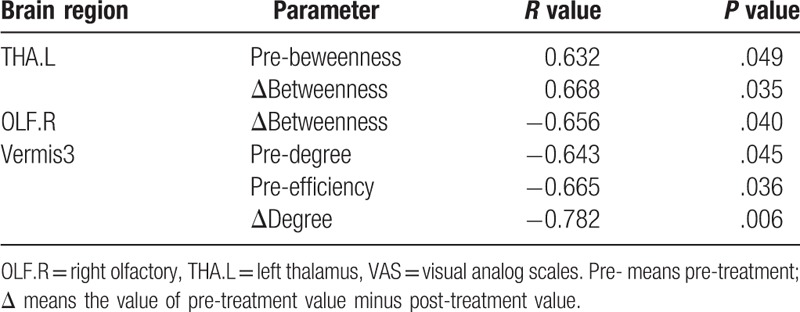
Correlation of node-level parameters with ΔVAS (*P* < .05).

**Table 4:**
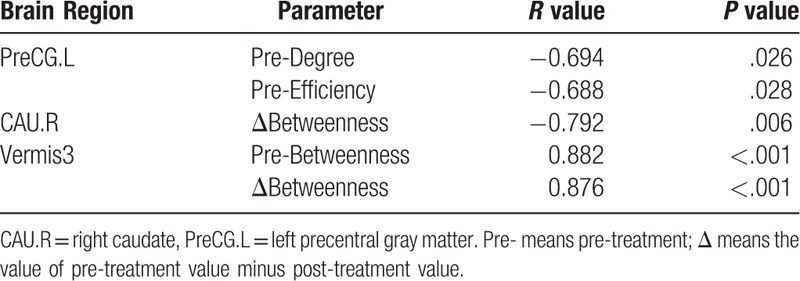
Correlation of node-level parameters with pre-treatment pain duration (*P* < .05).

## 4 Discussion

In this work, the effects of rTMS on the whole-brain functional network of PHN patients was explored by applying a series of network parameters. Firstly, the results showed that the global network parameters including characteristic path length, clustering coefficient, global efficiency, and local efficiency were changed at some specific network sparsity after rTMS. Secondly, the node-level network parameters including node degree, node betweenness and node efficiency were changed in some specific brain regions after rTMS. Thirdly, the node-level parameters showed significant correlations with clinical variables including VAS, ΔVAS, and pain duration.

### 4.1 Effects of rTMS on functional network global characteristics

In general, the brain functional network has a small-world architecture with high local clustering coefficient and small characteristic path length, which maintains the balance between the brain functional segregation and integration.^[14–17]^ The present results revealed no changes in small-worldness after rTMS, indicating that the whole brain network might keep a stable functional communication condition even after external magnetic stimulation. Our finding is in concordance with a previous study which found no small-worldness changes in the PHN patients compared to normal controls.^[4]^ Following rTMS treatment, the characteristic path length, which represents the long-range information transferring efficiency between distant brain regions, showed a significant increase at a few specific network sparsity. Besides, the clustering coefficient and local efficiency, which measures the connection density and shot-range information transferring of local neighboring brain regions, showed significant decrease at a large sparsity range (0.32–0.5 and 0.46–0.5, respectively). These alterations might be caused by the underlying suppression of local networks such as motor-related and pain-related modulation circuitries by rTMS. It might also disturb the local connection of other networks (such as emotion, cognition, and affective/memory), leading to a weak increase in local characteristic path lengths and a decrease in local clustering coefficients. Because the global and local efficiencies are consistent with the reverse of characteristic path length and the local efficiency is closely related to cluster coefficient, the global and local efficiency changed in a decreasing pattern. Note that there were 50 levels of network sparsity, among which only two showed significant changes in characteristic path length and global efficiency. Despite FDR correction for multiple comparisons, these significant values are still likely to be false positive. The results indicated that rTMS may primarily modulate the local functional circuitries with very little influence on the whole brain network architecture in PHN. This finding is in line with a previous report that found preferential changes of local node interactions in chronic pain.^[18]^

### 4.2 Effects of rTMS on functional network node-level characteristics

A node degree is defined as the total weighted value of direct connections between the present node and all the other nodes in a network. Node betweeness is defined as the ratio of the shortest information transferring path number between any other two nodes that pass through the present node and the total shortest information transferring path number between the two nodes. Node efficiency is defined as the averaged value of the reciprocal of the distance between the present node and the other node in a network. The larger the node degree, the more the edges connecting to the node, and the more important the node in the network. The larger the node betweenness, the shortest are the paths that pass through the node, the more important the node is as an intermediate node in the network. The larger the node efficiency, the faster the information is transferred between the node with other nodes in the network. All these parameters describe the importance of the node in the information flow of the whole network.^[19–21]^

There is no doubt that the node-level parameters within the sensory-motor network would change, because the stimulation was aimed at M1, mainly located at precentral gyrus, which is the hub of the sensory-motor network.^[22,23]^ This area is mainly related to motor execution and proprioception and is also involved in chronic pain processing.^[3]^ In fact, after rTMS, the bilateral precentral gray matter (PreCG) detected by node degree and efficiency showed a decreasing trend, which is most probably associated with the direct effect of 5-Hz rTMS. Even though the 5-Hz rTMS is thought to excite the neuron activities in the M1, it might also cause inhibition of the cortical neurons. The magnetic field can be manipulated by varying pulse intensity, frequency, duration, and the interval between pulse trains as well as by varying brain biochemistry and genetic background of the individuals.^[24]^ So, 5-Hz TMS stimulation effect (activation or inhibition) is still in debate in the clinical world.^[25]^ In this work, the effects of rTMS tended to inhibit the neurological activities within the M1 area. SMA.L (by node degree) and PCL.L (by node degree, betweenness and efficiency), which are related to the motor execution and somatosensory, showed decreasing trends after rTMS. There could be two possible reasons explaining these decreasing patterns. One possibility could be that as both the regions are spatially close to M1, they might be modulated directly by rTMS. The other possibility could be that both regions may be indirectly influenced through the sensory-motor network. THA is in the cortical-striatum-thalamus pathway and previous works have shown that the dural MCS (motor cortex stimulation) caused an antidromic activation of the thalamocortical pathway.^[26]^ Therefore, decreasing node betweenness of the left THA might be indirectly modulated by the stimulation of the M1 area. The THA is the information integration center for pain processing, and the decreased node betweenness after rTMS indicates that its intermediary role in the functional connection is weakened affecting the pain-processing pathway (thalamus- somatosensory cortices) which relieves the pain intensity.^[4,8]^ The cerebellum is in the cortical-pons-cerebellum pathway, and could be indirectly affected by the M1 stimulation.

We observed decreases in node degree, betweenness and efficiency in vermis3 and vermis45, which are parts of the cerebellum. All these alterations might be due to the direct influence of rTMS or indirect influence through the motor pathway and sensory-motor network. Previous reports have shown that the MCS not only affected the sensory-motor network but, also, the structures involved in affective/memory, cognitive and emotional aspects of pain,^[6,27]^ supporting our results. In our work, we found similar results. Anterior cingulum gray matter (ACG) and para-hippocampal gray matter (PHG) belong to the limbic lobe, mainly related to emotion, affection and memory processing, and are the most reported areas in acute and chronic pain. These are the key functional areas in the pain-perception, emotion, and cognition processing.^[4,28]^ Amygdala (AMYG) (by node betweeness) belongs to the limbic system and was also reported to participate in emotional signal processing of chronic pain.^[8]^ Generally, pain can cause depression, anxiety and fear, and these negative emotions could contribute to pain intensity.^[6]^ Following rTMS, ACG (by node degree and efficiency), PHG (by node betweenness), and AMYG (by node betweeness) showed decreasing trends, which might indicate a relief of the negative emotions and affections such as anxiety and fear. SFGmed (by node betweenness) is not only related to motor-function (pre-motor cortex) but also related to emotion and mental activities. Therefore, the alterations in this areas may indirectly be affected by PreCG through their intrinsic functional connections, which might suppress motion function or negative emotions. Lingual (LING) which is spatially near the PHG and is related to visual processing as well as logistic analysis and visual memory, has rarely been reported to participate in the pain processing and might have no direct role in PHN pain. We inferred that the decreasing values of node bewteenness in this region might be due to the disturbed network related to pain affection/memory processing especially related to PHG. The function of this area in PHN pain or rTMS warrants further investigation. All the decreasing trends observed in this study indicate that rTMS treatment could modulate and affect the sensory-motor network, emotion, cognition and affective/memory circuitries through the stimulation of the primary motor cortex. But the most commonly reported area of primary somatosensory cortex,^[4,29]^ S1, located at the post-central gyrus, showed no significant alterations in this study. This might be due to that rTMS did not have a substantial effect on the pain-perception circuitry as most of the patients (6/10) showed small changes in pain intensity according to the VAS scores after rTMS. Self-reporting by the patients revealed that their sleeping quality was remarkably improved after rTMS, indicating that the suppressed function of the above specific nodes in the brain network may be beneficial for sleeping in PHN patients. This is further supported by previous study which showed the functional connectivity strength of insomnia decreased after clinical treatment.^[30]^ Further investigations with a longer treatment course or other processing means would better our understanding on this.

### 4.3 Correlation of network parameters with clinical variables

The pre-treatment node betweenness of TPOmid.R showed a positive correlation with pre-treatment VAS, which indicated that the pain intensity might influence the pain-related cognition and emotional activities of this region. The increase in node betweenness of this region indicated the improvement of cognition and emotional activities (such hedonics and reward circuitries) after rTMS. The pre-treatment betweenness and Δbetweenness of THA.L showed a consistent positive correlation with ΔVAS, which indicated that in this region, the larger the pre-treatment node betweenness, the more the VAS alterations might be observed after rTMS. The VAS alterations were consistent with the alteration of node betweenness, which indirectly reflected the key role of THA in pain-related processing. This was consistent with the significant decreased node betweenness of THA.L after rTMS. The Δbetweenness of OLF.R showed a negative correlation with ΔVAS. That is to say, with large VAS alterations, opposite changes in node betweenness in this region appeared. This indicated that rTMS improved the emotional or memory processing. It was consistent with the increasing node betweenness in OLF.R after rTMS. The pre-treatment degree and efficiency of PreCG.L showed negative correlations with pain duration, indicating that the function of PreCG.L might be suppressed during PHN pain processing. The changes in node betweenness of CAU.R showed a negative correlation with pain duration, indicating that the cognition deficit in the pain course and the node betweenness of this region increased after rTMS. That is, if pain duration is long, it is difficult to recover the cognition function. Pre-treatment node betweenness of vermis3 showed a positive correlation with pain duration due to its participation in the PHN pain processing. The reduction of vermis3 node betweenness after rTMS showed a positive correlation with pain duration. Specifically, if the pain duration is long, the intermediate role of vermis3 can change easily after rTMS. The above results indicated that the alterations of these regions could be used as neurological biomarkers for PHN and for rTMS treatment evaluation.

## 5 Limitations and future works

The main limitations of this work are:

1. small sample size; and
2. some brain regions are not fully understood and need further investigating.

For future study, a larger cohort of patients will be recruited and different methods will be applied and compared to validate the results. Besides, more types of pain diseases and treatment effects would be evaluated by using more advanced MRI techniques. In addition, other methods are available for functional imaging and should be explored in order to refine our understanding of PHN and rTMS. These methods include, but are not limited to, representing and retrieving video shots in human-centric brain imaging space^[31]^ and extendable supervised dictionary learning for exploring diverse and concurrent brain activities in task-based fMRI.^[32]^ Novel methods could also be applied from other fields and inspire novel imaging approaches to evaluate the effects of rTMS in PHN patients.^[33,34]^

## 6 Conclusion

rTMS has significant effects on the regional homogeneity of PHN patients, with a potential for suppression of sensory-motor function and improvement of emotion, cognition, affection, and memory function. Furthermore, these alterations in the regional homogeneity might be used as neural biomarkers for rTMS post-treatment evaluation.

## Author contributions

**Conceptualization:** Jiaxiang Ni.

**Data curation:** Bin Jing.

**Investigation:** Qianqian Meng.

**Methodology:** Xiangyu Ma.

**Resources:** Xiao Mo.

**Software:** Zhizheng Zhuo.

**Supervision:** Han Liu.

**Validation:** Wei Liang.

**Visualization:** Wei Liang.

**Writing – original draft:** Qian Pei.

**Writing – review & editing:** Haiyun Li.
